# Recruiting a Hard-to-Reach, Hidden and Vulnerable Population: The Methodological and Practical Pitfalls of Researching Vaccine-Hesitant Parents

**DOI:** 10.1177/10497323231196439

**Published:** 2023-09-06

**Authors:** Ana Patrícia Hilário, Alice Scavarda, Dino Numerato, Joana Mendonça, Mario Cardano, Jaroslava Marhankova, Luigi Gariglio, Pia Vuolanto, Alistair Anderson, Petra Auvinen, Piet Bracke, Tom Douglass, Pru Hobson-West, Esther Lermytte, Paulina Polak, Tadeusz Rudek

**Affiliations:** 1 37809 Instituto de Ciências Sociais da Universidade de Lisboa, Lisboa, Portugal; 2 Dipartimento di Culture Politica e Società, 9314 Universita Degli Studi Di Torino, Torino, Italy; 3 Fakulta sociálních, 37740 Univerzita Karlova, Praha, Czech Republic; 4 Faculty of Social Sciences, 7840 University of Tampere, Tampere, Finland; 5 School of Sociology and Social Policy, 6123 University of Nottingham, Nottingham, UK; 6 Department of Sociology, 26656 Ghent University, Gent, Belgium; 7 Department of Social Work & Social Care, 1724 University of Birmingham, Birmingham, UK; 8 Instytut Socjologii, 37799 Uniwersytet Jagiellonski, Krakow, Poland

**Keywords:** vaccination, trust, recruitment, hesitancy, interviews

## Abstract

While recruitment is an essential aspect of any research project, its challenges are rarely acknowledged. We intend to address this gap by discussing the challenges to the participation of vaccine-hesitant parents defined here as a hard-to-reach, hidden and vulnerable population drawing on extensive empirical qualitative evidence from seven European countries. The difficulties in reaching vaccine-hesitant parents were very much related to issues concerning trust, as there appears to be a growing distrust in experts, which is extended to the work developed by researchers and their funding bodies. These difficulties have been accentuated by the public debate around COVID-19 vaccination, as it seems to have increased parents’ hesitancy to participate. Findings from recruiting 167 vaccine-hesitant parents in seven European countries suggest that reflexive and sensible recruitment approaches should be developed.

## Introduction

Taking research conducted with vaccine-hesitant parents as a starting point, this article discusses the main challenges involved in recruiting a hard-to-reach, hidden and vulnerable population, and it provides some general insights to studies addressing populations with similar characteristics. Previous research suggests that when vaccine-hesitant parents avoid participating in research, it might be due to the sensitivity of the matter under study (Ellard-Gray et al., 2015; Shaghaghi et al., 2011). These parents may not want to openly discuss their choices about vaccination due to the risk related to self-disclosure (Sydor, 2013) and thereby with their association with ‘anti-vaxxers’. Research suggests that this association often leads these parents to experience stigma and discrimination (Carpiano & Fitz, 2017; Wiley et al., 2021). This experience was accentuated by the COVID-19 pandemic as there has been an intensification of the ‘demonization’ of individuals who oppose to vaccination in public debates (Jaspal & Nerlich, 2022) and a polarization of the debate regarding vaccination (Mønsted & Lehmann, 2022).

Although a few studies explicitly reflect on the difficulties concerning the sampling of vaccine-hesitant parents (e.g. Reich, 2015; Ward et al., 2017; Wiley et al., 2020), to our knowledge none of these accounts understood vaccine-hesitant parents as a hard-to-reach, hidden and vulnerable population. The need for this conceptualization emerged as an outcome of our research carried out among vaccine-hesitant parents in seven European countries and, in particular, as part of the continuous reflexivity of our fieldwork activities. Vaccine-hesitant parents may be difficult for researchers to recruit because (i) they do not share a physical location nor are organized in visible groups which could be directly contacted (hard-to-reach); (ii) there are no records of vaccine-hesitant parents and they may not wish to be contacted or found due to their identity as well as due to the related risk of being exposed to sanctions especially in national contexts with compulsory vaccination systems (hidden); and (iii) they are potentially at risk of suffering stigmatization or of being discriminated by others because of their choices or opinions regarding vaccination (vulnerable). A recent review highlighted the importance of qualitative work to promote a more in-depth understanding of vaccine hesitancy (Dubé et al., 2016), while other work addressed several methodological issues (Dubé et al., 2018). However, in which the issues of access or recruitment were not systematically discussed. Against these circumstances, we provide a systematic methodological discussion to address the main aspects of qualitative research within this population. Thus, our contribution will provide a focus not apparent in the existing literature, about the challenges to the participation of vaccine-hesitant parents. The need to be reflexive (Lumsden, 2019) throughout the research process, including the recruitment phase, is key for the successful completion of the research.

### Defining Vaccine-Hesitant Parents

In any research project, the first step is to try and identify participants (Patel et al., 2003); therefore, it is crucial to identify who may be placed under the umbrella of ‘vaccine-hesitant’. The term vaccine-hesitant parents broadly refers to parents who may either delay or refuse the vaccination of their children as recommended by local health authorities or express doubts and concerns about it. Although the most prominent definition of hesitancy is provided by the Strategic Advisory Group of Experts (SAGE) on Immunization and focuses on behaviours, namely practices of delaying or refusing vaccines (MacDonald and the SAGE Working Group on Vaccine Hesitancy, 2015), other scholars distinguish between practices and motives of concern (Benin et al., 2006), highlight the gap in parental knowledge (Rees & Madhi, 2011) or reflect on the benefits of specific vaccines (Velan, 2011).

Furthermore, scholars have identified some tensions in the way the term is being used. For example, Bedford and colleagues argue that ‘(1) “Vaccine hesitancy” is represented as a behaviour, even though it is a psychological state; (2) the label “hesitancy” is applied to non-vaccination broadly, when in fact some non-vaccinators are forthright in their refusal, and may have never been hesitant; and (3) “hesitancy” is used inaccurately as the explanation for under-vaccination in a population when the causes are related to pragmatics, competing priorities, access, or failure of services or policies’ (Bedford et al., 2017, p. 1).

More recently, the WHO defined hesitancy as a ‘motivational state of being conflicted about, or opposed to, getting vaccinated; includes intentions and willingness’ (2022, p. ii). Vaccine-hesitant parents are therefore seen as somewhere on the continuum between acceptance and refusal of vaccines, but what falls under this umbrella term is disputable (Dubé et al., 2021). Rather than a continuum, others have focused on the way in which hesitancy combines different factors. For example, Peretti-Watel and colleagues refer to vaccine hesitancy as a combination of beliefs, attitudes and behaviours, both considering parents who reluctantly conform (who may accept immunization despite their doubts) or adopt vaccine-specific behaviours (Peretti-Watel et al., 2015). Therefore, the current study follows Wiley and colleagues’ (2020) recommendations of a ‘nuanced personalized engagement with non-vaccinating parents’ rather than a ‘one-size-fits-all approach’ (2020, p. 2).

## Background

While the need to develop qualitative studies for a better understanding of vaccine hesitancy has been acknowledged (Dubé et al., 2016), the process of recruiting vaccine-hesitant parents into qualitative research has not been deeply discussed (Reich, 2015). The continuous reflexivity of the fieldwork activities performed by each research team across different sites further enhanced our awareness of hesitant parents as a hard-to-reach, hidden and vulnerable population. The limited literature available suggests that the sampling of these populations should be an iterative process, as researchers learn how best to meet the needs of these populations along the research (Ellard-Gray et al., 2015). These populations are often invisible (Faugier, 1997) and may want to conceal their characteristics and behaviour and, consequently, may not easily agree to cooperate in a study (Shaghaghi et al., 2011).

Some studies have reported challenges in recruiting vaccine-hesitant parents specifically due to issues of trust; however, this has not been considered in significant depth (Wiley et al., 2020). Indeed, mistrust in the research activities due to a critical stance related to the academic process (Sutherland & Fantasia, 2012) has been a well-documented barrier to participation (Bonevski et al., 2014). The latter includes concerns about the usefulness or the potential harm produced by the research findings (Ellard-Gray et al., 2015) to the community. This may be particularly relevant when studying vaccine-hesitant parents due to their reported general scepticism towards science (Kate et al., 2021). The degree of consent to participation depends on the characteristics of the group, on the recruitment method, and on the specific research circumstances – including cultural, social and economic barriers – as well as on specific reasons for participating in research (Sydor, 2013).

The establishment of a sampling frame of these parents might be challenging due to their reluctance to self-identify as vaccine-hesitant (Condon et al., 2019). This reluctance could be related to perceived or experienced feelings of stigmatization (Wiley et al., 2021) or social pressures perceived from other members of the community, particularly because their behaviours are not compliant with the social norms (Shaghaghi et al., 2011) which prescribe vaccination. Although scholars suggest diversifying recruitment strategies, by mixing their advantages and limitations, their success seems to be largely due to researchers’ knowledge of the population and ability to flexibly adapt to it (Shaghaghi et al., 2011). Indeed, recruitment has been acknowledged as a time-consuming task that may involve extra effort on the part of researchers (Thomas et al., 2007). This may be particularly challenging when recruiting hard-to-reach, hidden and vulnerable populations. These populations share the same characteristics: ‘(a) non-existent sampling frames and thus the size of the membership and group boundary is unknown; (b) acknowledgment of belonging to the group is threatening, as membership involves being the object of hate or scorn and sometimes fear of prosecution; and (c) members are distrustful of non-members, doing whatever they can to avoid revealing their identities, and are likely to refuse to cooperate with outsiders’ (Benoit et al., 2005, p. 264).

While some studies in the field of social sciences have specifically addressed the challenges of recruiting hard-to-reach (e.g. Chiang et al., 2001), hidden (e.g. Hardwood et al., 2012) and vulnerable populations (e.g. Liamputtong, 2007), these issues have not been deepened in relation to their participation in research by vaccine-hesitant parents. Drawing on a project based in seven European countries, we contribute to fill this gap by discussing the recruitment strategies used with vaccine-hesitant parents, as well as acknowledging the barriers encountered in recruiting this specific population.

## Methods

### The Study

The paper is based on the findings of an international team ethnography (Erickson & Stull, 1998), carried out in 2022 within the VAX-TRUST project in seven European countries: Belgium, Czech Republic, Finland, Italy, Poland, Portugal and the United Kingdom. These countries have significant differences in size, vaccine coverage and healthcare systems. Moreover, in three of these countries, there are policies making immunization compulsory (Czech Republic, Italy and Poland), and in two of them (UK and Finland) immunization is highly recommended. Belgium and Portugal are in an intermediate position, with some vaccinations compulsory and others recommended. Whereas fieldwork was performed differently in each country due to cultural and contextual differences, the approach and the design of our study shared a common framework.^1^

In each national context, researchers conducted in-depth interviews with HCPs and vaccine-hesitant parents, as well as carried out participant observation in healthcare sites (i.e. healthcare centres, pediatricians, GP surgeries, hospitals and children’s agencies) to have access to both practices and discourses related to hesitancy, in line with the theoretical framework of the study. Moreover, they were useful to tackle the factors that impact vaccine hesitancy, as they allow the observation of interactions, and to gain participants’ representations. For the purposes of this paper, we will focus on the recruitment process for interviewing vaccine-hesitant parents.

### Data Collection Procedure

Although many parents refused to participate in our study, we were able to conduct 158 interviews (either individually or with couples) in the seven European countries of the consortium. A total of 167 parents who have a child aged 6 years or under and have delayed or refused at least one compulsory or recommended vaccine were interviewed. There were some differences between countries due to recruitment challenges. In a few of the studied contexts, parents who had doubts and concerns about vaccination, although having children fully vaccinated, were also considered eligible to enrol in the study (they were defined as ‘concerned compliant’).

Most interviewees were mothers, and only in a few cases interviews were conducted solely with fathers (*n* = 13) or with couples (*n* = 9). Regardless of their gender, interviewees had an average age of 37.9 years. Initially, our research design was planned exclusively to include face-to-face interviews. However due to the COVID-19 pandemic, we gave parents the possibility to conduct an interview either face-to-face or via an online platform. Online interviews offered the parents the opportunity to be interviewed in a safe and private space, without the pressure to secure childcare (Varma et al., 2021). In addition, it helped to reach parents who lived outside of large urban centres, in rural areas, as well as those who would, otherwise, be unavailable. Furthermore, it also enabled us to conduct interviews during a pandemic period when travel was restricted, access to homes was limited and it was not possible to meet in public spaces.

A detailed description of the sample of the present study is provided in Table 1. All ethical aspects of the project were managed, monitored, reviewed and approved by the transnational Ethics Advisory Board. Ethical approval was granted in each country.

**Table 1.** Description of the Sample by Country.

Country	Number of interviews	Inclusion criteria	Exceptions/particular cases	N	Interviewee: mother	Interviewee: father	Interviewee: couple	Mean age	In presence	Online	Interviews’ length (mean) (in minutes)
Belgium	15	• Worrying about your children’s vaccinations	• One mother with the youngest child being 12	16	14	-	1	39	3	12	54
		• Refusing certain vaccines	• One mother with the youngest child being 11								
		• Postponing certain vaccines	• One mother with adult children								
		• Having (at least) one child									
		• Youngest child being 7 or younger									
Czech Republic	30	• Having intentionally postponed or refused at least one of the compulsory vaccinations	-	30	30	-	-	35	10	20	50
Finland	24	• Having a child aged 18 years or under	• Four interviewees with adult children and one childless interviewee	25	21	2	1	43	6	18	54
		• Having delayed or refused at least one vaccine or having doubts and concerns about vaccination									
Italy	23	• Having delayed or refused at least one vaccine or having doubts and concerns about vaccination	• The case of a couple who had a child aged 8 years and decided to get only the hexavalent vaccine	30	15	1	7	39	8	15	90
		• Having a child aged 6 years or under									
Poland	24	• Living in the target region	-	24	22	2	-	-	22	2	27
		• Having delayed or refused at least one vaccine or having doubts and concerns about vaccination									
		• Having a child aged 6 years or under									
Portugal	31	• Having a child aged 6 or under	• In some cases (*n* = 10), parents with children over the age of six were also interviewed	31	28	3	-	39.6	-	31	63
		• Having delayed or refused at least one recommended vaccine									
United Kingdom	11	• Living in the target region	-	11	6	5	-	32	-	11	28
		• Having a child under 6 years									
		• Having ticked disagree or totally disagree to a screener Likert question set by Prolific: ‘I believe that scheduled immunizations are safe for children’									

In what follows, we provide a reflexive account of the recruitment process. Empirical evidence consists of fieldnotes, reflexive research diaries and research project meeting’s minutes during which we have progressively reflected upon the recruitment process, notably its opportunities and challenges and newly emerging sampling strategies. In other words, here we do not analyze data from 158 interviews, but we critically reflect upon the journey that allowed us to conduct them.

### Recruitment Strategies

In all countries, the strategies adopted were based on our understanding of the likely profile of vaccine-hesitant parents, based on the results of previous publications and our own fieldwork. For example, former research on vaccine hesitancy (see, for instance, Dubé et al., 2013) highlighted that hesitant parents sometimes combine allopathic and complementary therapeutic practices and favour consumption patterns related to organic or natural living. We, therefore, preliminarily, searched for parents of young children who adopted the so-called ‘alternative’ lifestyles, referring mainly to natural child rearing practices. However, in the end, we developed plural strategies to increase the heterogeneity of our samples, and not to limit the recruitment only to this category. Several recruitment channels, therefore, were used to reach vaccine-hesitant parents. These included the use of social media, one subject referred other subjects (i.e. snowballing as proposed by Atkinson and Flint (2001)), boards of ‘alternative’ schools, personal contacts, university mailing lists, organizations focusing on parenting, direct invitations, in-person selection at vaccination sites, local associations and mediators, informational flyers and posters and, in the case of the United Kingdom, a research recruitment platform called Prolific.

## Results

While we anticipated the difficulties we could have faced as part of the recruitment process, it was only during our fieldwork that we realized that vaccine-hesitant parents represent a hard-to-reach, hidden and vulnerable population. Vaccine-hesitant parents were a challenge to recruit. In what follows, we provide examples of our experiences which show why vaccine-hesitant parents should be considered a hard-to-reach, hidden and vulnerable population. It is important to stress that not all the recruitment strategies were attempted in all countries; however, the breadth of evidence provides valuable insight into the experience of conducting research in this area. For instance, in the United Kingdom, the recruitment strategy was mainly based on the Prolific platform, albeit several attempts were made initially to use other channels. The focus of the current paper will be to reflect on the challenges faced to recruit vaccine-hesitant parents, and thereby the data presented will not focus on the interviews per se but instead will be limited to the interview recruitment process.

## Hard-to-Reach Population

Vaccine-hesitant parents are a hard-to-reach population due to their complex and multifaceted characteristics; therefore, no sampling frame is available or can be defined. Some of the existing studies recruited vaccine-hesitant parents through associations established by vaccine-hesitant parents, or parents opposing vaccination (Hobson-West, 2007; Numerato et al., 2021). This was also applied in our study. Not all strategies worked in all countries, but overall, participants were successfully identified based on contacts with non-governmental organizations focused on parenting (e.g. maternity care). These contacts were made through adverts published in newsletters and on those organization’s web pages. University students, namely those using services for parents, were also reached through adverts distributed via a mailing list, newsletters and intranet. Cultural mediators helped to establish contact between researchers and local associations. This enabled us to recruit parents in long-term settled communities. Nevertheless, it should be pointed out that community immersion was not possible in most countries for recruiting vaccine-hesitant parents.

Some research to date suggests that vaccine-hesitant parents may opt for alternative lifestyles which may compromise non-mainstream educational models (Byström et al., 2014; Sobo, 2015). Therefore, in some countries, boards of schools or kindergartners such as Steiner, Forest and Waldorf, as well as others following the Anthroposophical movement, were contacted either by phone or by a descriptive email with an invitation to disseminate the project through parents’ mailing lists. Nevertheless, almost no heads of alternative schools replied to the invitation or agreed to collaborate in the research. In some contexts, this happened even notwithstanding the previously existing relationships of trust between the representatives of alternative institutions and team members. While we had some idea about the vaccine-hesitant approach in the context of alternative education, the silence itself represented one of the proofs of the fact that vaccine-hesitant parents represent a hard-to-reach population. Those who responded to the invitation, but refused to disseminate the study, argued that they were not allowed to ask parents about their children’s immunization practices. Other school boards explained that they refused to participate on the grounds that they did not want to enlarge the existing polarization of attitudes between parents.

Informational flyers and posters were distributed in places frequently visited by young parents such as hospitals, primary schools, day cares and alternative medical practices. For instance, flyers containing a description of the project and an invitation to participate in the study were distributed in sites related to alternative lifestyles, such as CAM (Complementary and Alternative Medicine) practices, organic food supermarkets, toy shops based on alternative educational models (e.g. Montessori) and natural products’ pharmacies. Nonetheless, the distribution of flyers did prove to be a successful strategy in some countries, allowing a direct link between research members and parents who, thus, might have perceived a safer space for interaction, with no intervention from intermediaries. These various strategies we needed to test and use to obtain participants during our data collection demonstrate that vaccine-hesitant parents are indeed hard to reach.

## Hidden Population

Vaccine-hesitant parents may not wish to be contacted or found. The use of social media appeared to be the best solution to find this hidden population. Therefore, in some countries, adverts on Facebook were published aiming to publicize the study and invite parents to participate. The adverts were published in either open or closed Facebook communities, some focusing on natural birth, extended breastfeeding, parenthood (e.g. alternative parenting style groups such as anthroposophical parenting) or vaccine critics (e.g. COVID-19 hesitant groups) and some focusing more broadly on community care, natural lifestyles or parenthood. Some parents were directly invited by email or through their Facebook profile (e.g. being a doula) due to the fact that they were part of alternative lifestyle communities, or after they had contacted the researchers themselves. Closed groups in the social network Telegram were also approached. In most countries, we intentionally avoided vaccine-critical groups (Hobson-West, 2007), given concerns that this may lead to the identification of more active individuals. Another challenge in the recruitment process was engaging with community mediators in the research, such as parents who were responsible for some vaccine-hesitant WhatsApp or Facebook groups. For instance, the leader of a WhatsApp group replied that she did not consider our study impartial even though most of our team members are social scientists, due to the fact that a few of them have a background in public health or health sciences’ areas. Furthermore, some parents detailed that they have gotten into trouble by disseminating our study. This was the case of a mother who posted an advertisement on a Facebook group of parents (not directly related to vaccination) and acknowledged that ‘There was a heated discussion when I made the post. Some of the parents quite sharply criticized me for posting such things, as such research is just a way of manipulating the parents’ (extract from fieldnotes).

In one country (United Kingdom), where other recruitment strategies did not work within the timescale, recruitment switched to the use of a research recruitment platform. This online platform connects researchers to potential participants based on predefined demographic characteristics such as being a parent, living in the target region and having a child under 6 years of age. Additionally, participants were also targeted based on their responses to a screener Likert question set, already included in the Platform: ‘I believe that scheduled immunizations are safe for children’.

In addition, parents were also recruited during participant observation in vaccination centres. The teams adopted a sort of in situ recruitment (Cicourel, 1964) by selecting some parents based on their displayed hesitant behaviours. Recruiting parents during observations was useful to persuade them to participate, in the sense that they become – at least tepidly – familiar with researchers, fostering a sense of trust. Nevertheless, this was not a successful strategy in all countries. The multiple forums of recruitment we used highlight that this population is hidden.

## Vulnerable Population

Vaccine-hesitant parents tend to experience or to perceive stigmatization (Wiley et al., 2021) or experience social pressures from other members of the community, particularly because their behaviours are not compliant with the social norms (Shaghaghi et al., 2011) that prescribe vaccination. Therefore, they can be considered a vulnerable population. Snowballing appeared to be a good strategy for the recruitment of vaccine-hesitant parents as it has been previously used in similar studies (e.g. Ward et al., 2017; Wiley et al., 2020). This strategy was adopted through different means: recommendations (i) by researchers who have previously developed research in natural birth or natural motherhood; (ii) by other parents who have also participated in the study; (iii) by healthcare professionals; and (iv) by researchers’ family members, friends, co-workers or other pre-existing contacts. In a similar way to Reich (2016), we found that parents had difficulties in recommending other parents to participate in the study. That said, while snowball sampling can contribute to the recruitment process, it cannot be used in isolation, as a unique recruitment tool. When parents were asked why they decided not to participate in the study, their answer appeared to be very much related to lack of trust: (i) in the VAX-TRUST project; (ii) in researchers (iii) in institutions; (iv) in the funding body; and (v) in public health authorities. Indeed, when invited to participate in the study, these parents expressed their suspicions about the aims of the project as they were afraid that the results of the study would be used to convince other parents to vaccinate their children. For instance, a mother explained to researchers why she would not be involved in research that could serve to persuade ‘the likes of me’ (extract from fieldnotes). Some parents were sceptical about the credibility of researchers as, according to them, they did not have independence from their funding bodies. Moreover, some of these parents believed in conspiracy theories regarding vaccination. For instance, some parents refused to participate in the study because they believed that the results would be manipulated by the European Commission (i.e. the funding body) or by national public health authorities, or that these are corrupted by the collaboration with entities that bear with them business logic (companies). A member of a vaccine-critical group on social media strongly recommended others not to participate in our research because the research receives funding from the European Commission; due to the fact that this and other comments were not immediately responded by the researcher, the administrator decided to remove the research announcement from the social media group. On reflection, the first version of the website of our study may have enhanced these parents’ suspicions, due to the way in which the goals of our research were initially presented. For example, in the first version for the project’s website text, we started out by using expressions like ‘dealing with’ vaccine hesitancy, which could be interpreted as an intention to ‘getting rid of’ or ‘handling’ people with vaccine-hesitant perceptions, which was, of course, not at all our intention. We rephrased this with expressions like ‘understanding’ vaccine hesitancy to be more precise in rhetorical terms. Also, we specifically avoided expressions with connotation with conflict or even war, such as ‘tackle’ or ‘combat’ in order not to give the impression that we would be against vaccine-hesitant individuals as such, or that vaccine hesitancy does not deserve a place in the public discussion (Goldenberg, 2016, 2021).

Other parents mentioned the ‘nonsense’ of vaccinations, referring the researcher to literature he/she regarded as ‘fundamental’ to understanding how vaccines are meant to do more harm than good. This suspicion was augmented by the COVID-19 pandemic as, according to their beliefs, the vaccine against the novel coronavirus was dangerous to human health and was created as part of a bigger plan by the European Union to control the population. The public debate around COVID-19 has further stimulated the polarization on vaccination, as well as the perceived stigmatization of vaccine-hesitant people (Bor et al., 2023). For instance, a mother refused to advertise our project by saying, ‘There is so much controversy around vaccination right now (in the context of the COVID-19 pandemic), and I don’t want to be publicly associated with this topic. I can share the information about your project with some of my close friends. However, I’m not willing to post it publicly on my profile’ (extract from fieldnotes).

Additionally, on a more practical level, the COVID-19 pandemic has undermined the availability of potential research participants, who were understandably focused on managing other health, personal or work-related issues arising from the pandemic. Vulnerability is evident here, and it gets combined with reluctance to collaborate with certain societal intervenients. Nevertheless, some parents disclosed that they wanted to participate in the study to contribute to a limited field of research and to express their opinion publicly.

## Discussion

Our critical reflections on working on a large international study suggest that vaccine-hesitant parents are a hard-to-reach, hidden and vulnerable population for several reasons. These include their potential vulnerability, in being exposed to sanctions, subjected to discrimination or stigma and their relative lack of organization, making identifying the relevant population a challenging task. Previous research had recruited vaccine-hesitant parents mainly through online communities where discussions on vaccination were common (Reich, 2020). Indeed, the advertisement in Facebook’s natural parenting groups has been previously described as a successful recruitment strategy (Wiley et al., 2020). However, given the risk of polarization and the potentially controversial nature of the vaccination topic in the public debate in the post-truth context (Numerato et al., 2019), Facebook and other online tools needed to be used carefully and thoughtfully. While online sites may help to enhance the recruitment process, they can also notably hinder it once the nature of the project or its funding body starts to be problematized. Using online recruitment requires a readiness from the researchers to sensitively and quickly react to the comments that their invitations to participate in research can foster (Reich, 2015). Furthermore, snowball and convenience sampling have been used to recruit vaccine-hesitant parents (e.g. Deml et al., 2021; Popper-Giveon & Keshet, 2022; Ward et al., 2017; Wiley et al., 2020). Indeed, this is aligned with the findings of the current article in which we point out the need to use snowball sampling as one of several tools rather than as the only isolated tool. Unlike what has been described in the literature (Wiley et al., 2020), advertisement in schools associated with the Anthroposophical movement did not prove to be a successful strategy in the countries where this was attempted.

The difficulties in reaching vaccine-hesitant parents were related to issues concerning trust, as there appears to be a growing distrust in experts (Vuolanto et al., 2020) which is extended to the work developed by researchers and their funding bodies. Indeed, mistrust in research or researchers has been documented in literature as one of the major barriers to the recruitment of hard-to-reach populations (Bonevski et al., 2014). Trust depends not only on the direct interaction between researchers and participants, as well as indirectly, through reputational effects not only related with researchers but with their funding bodies (Celestina, 2018). Although parents do not directly express concerns about the usefulness, or the eventual harm produced by the research findings, they were suspicious about the aims of the researchers and the use of their results. The suspicion that vaccine-hesitant parents tend to have towards researchers has been previously acknowledged (Reich, 2015). Indeed, research suggests that vaccine-hesitant parents are highly critical of the information given by institutional science (Kate et al., 2021). These difficulties have been accentuated by the public debate around COVID-19 vaccination, in that this may have increased parents’ reluctance to participate in research. The comments made by some potential participants during the recruitment phase had an emotional impact on researchers in some countries. This was very much related to participants questioning the impartiality of researchers, accusing them of being part of a conspiracy developed by the funding body, for example, to control population using vaccination. This was stimulated by the fact that some members of our project team, although not necessarily being involved in the interviewing process, had a public health background. In the broad collaboration, we had the asset of sharing experiences from difficulties and successes in recruitment and gathering ideas and perspectives across different teams in various countries. This enabled learning from each other and peer support when undergoing the recruitment processes, simultaneously, in the different countries.

The purpose of our interviews was to ‘hear about parents’ views and opinions, and not to ‘test’, judge, legitimize or commend them or their decisions’ (Kate et al., 2022, p. 3). While researchers were aware that because of their academic background (i.e. social scientists) and experiences (e.g. being a mother/father of children under 6 years old, or a parent who had decided to accept vaccination) they had a personal position about the phenomenon under study, they made a strong effort to ensure a space of openness, rapport and confidentiality to participants (Lawrence, 2022). Trust, rapport, empathy and understanding have been found to be key in developing research on sensitive matters (Bahn & Weatherill, 2012). As social scientists, we therefore aimed to operate in a non-judgmental manner, in relation to multiple perspectives towards vaccination. In some cases, this seemed to be appreciated by participants. For example, one interviewee reflected afterwards that the interview experience was ‘healing’ for her because she did not feel stigmatized as a ‘wingnut’ or a ‘tin foil hat’ due to her vaccine-hesitant thoughts (extract from fieldnotes). In our study, the researchers’ positionalities (e.g. gender, age and being a parent of a child or not) were sometimes acknowledged as a way of promoting trust and empathy with participants. On the one hand, researchers with young children were more likely to empathize with interviewed and observed parents. On the other hand, this sometimes required the researchers to engage in emotional labour, as these parents’ beliefs and behaviours may not always be in accord with those of researchers regarding childhood vaccination. When this was the case, researchers tried their best to ‘manage “doing similarity” with participants’ (Reich, 2015, p. 403). The emotional reactions that emerged, either in formal or informal conversations with participants, were used to better understand the phenomenon under study (Rodríguez-Dorans, 2018). Through reflexivity, researchers became aware of how they were understood by participants (Takeda, 2021). Reflexivity was thus an important methodological tool as it enabled us to acknowledge the subjectivities of both participants and researchers as their positionality and biography may have had an impact on the research process (Borgstrom & Ellis, 2021).

The COVID-19 pandemic instigated the development of creative solutions to recruit vaccine-hesitant parents (Archer-Kuhn et al., 2021) such as online recruitment through social media platforms. Overall, our research experiences highlight the importance of adopting flexible and sensible approaches for recruiting hard-to-reach, hidden and vulnerable populations in different contexts (Bamidele et al., 2018; Condon et al., 2019) as well as the importance of using multiple recruitment procedures (Ramlagan et al., 2021).

The importance of building trust with participants through community immersion, in this case participant observation in vaccination contexts, is confirmed as a factor influencing the willingness to participate in research (Linders & Chifos, 2018). Considering that one of the major barriers to participation in the current study was mistrust in the researchers, one of the most successful strategies to overcome this was to address participants’ concerns and suspicions and to reassure them that we would like to listen to their experiences in a non-judgmental manner. In addition, mistrust appeared to be augmented by the COVID-19 pandemic and the polarization of the speeches around vaccination. Whereas some parents were worried about being labelled as ‘anti-vax’, others considered themselves as ‘freethinkers’ and did not want to be involved in research funded by institutions such as the European Commission. Non-participation in research may, to a certain extent, be understood as an everyday resistance, with the aim of undermining the power of certain institutions (Vinthagen & Johansson, 2013). Indeed, vaccine refusal is for some parents: ‘a highly social act – an act that, each time it is undertaken, reinforces social belonging by vitalizing community ties’ (Sobo, 2016, p. 345). Furthermore, our findings suggest that the lack of trust by vaccine-hesitant parents in institutions or experts (Kate et al., 2021) is extended towards researchers; according to their opinion, *we* are part of the *system*. In a similar way, scepticism towards science and scientists has been studied in relation to elite populations (Ostrander, 1993). Indeed, our sample confirms that vaccine-hesitant parents tend to have a higher social capital which ‘create and maintain subcultural norms that contradict broader social norms and provide sources of individual support for doing so’ (Reich, 2020, p. 7).

In this context, recruitment needs to be understood as a process that requires interactive strategies and open space for communication. Static instruments such as advertisements or flyers have a lower probability of success, if not accompanied by tools to define, re-define or negotiate the perspective of researchers. More dynamic instruments, including social media or participant observations, may provide much needed communication space to build, nourish and maintain trust between researchers and research participants, to provide assurance, address their understandable suspicions or, eventually, to deconstruct interpretations and assumptions about the position of research. Trust could also be reinforced by highlighting the specificity of the qualitatively oriented understanding of social scientific research, as opposed to the way which the so-called ‘hard’ natural biomedical sciences are sometimes perceived.

Opening space for dialogue and interaction between participants and social scientists can maximize the potential of recruitment strategies. Moreover, the dynamic nature of the recruitment process was, in some national contexts, enhanced thanks to the use of ethnographic observations. In this regard, adopting the underused method of vaccine-hesitant ethnographic observations (Dubé et al., 2018) proved not only to be a tool for generating data but also a route via which it is possible to reach hidden respondents. Furthermore, in our experience, stressing that participation equated to an opportunity to share their point of view did serve to reassure some participants (Condon et al., 2019). While our reflexive account discusses a plethora of strategies to cope with hard-to-reach, hidden and vulnerable populations, we do realize that they inevitably face certain limitations. The meanings attributed to the funding body represented a structural challenge that we could only hardly bypass in our research endeavour. We acknowledged this aspect, in particular, when comparing this research study with research projects that several research members had previously undertaken. It is worth adding, however, that the previous studies had the disadvantage of not providing transnational comparative evidence. Moreover, we appreciate that not all possible research strategies have been utilized. For example, participant observations of vaccine-hesitant parents as researchers would allow for a more in-depth insight into the hidden nuances of the research field. Third, while the reflexive fieldnotes, research diaries and regular research meetings proved to be a valuable source of evidence to develop this reflexive account, the project-driven nature of the study, with strictly confined temporality, did not allow us a more continuous and systematic comparison, ex-ante designed reflexive observations. In a similar way to Kristensen and Ravn’s (2015) work, the findings of this article confirm the importance of researchers adopting a more subtle approach to recruitment. We followed the advice of McCormack and colleagues (2013) that researchers should be sensitive to the social context where the research takes place and be flexible to adapt the methodological approach. Therefore, reflexivity is key in the research process (Rossman & Rallis, 2010) as it enables researchers to deal with the practical issues (Roriz & Padez, 2017) that emerge during the recruitment of research participants (Dawson et al., 2017). Recruiting hard-to-reach, hidden and vulnerable populations calls for an extended notion of reflexivity (Lumsden, 2019). Lumsden (ibid) argued that reflexivity focuses on the unfamiliar, the uncomfortable, the messy, the difference and the importance of writing up researchers’ failures. A reflexive approach enables researchers to be conscious of the social, ethical and political impact of their research and of the changing nature of their power relations (with participants, cultural mediators, and research funders).

## Conclusion

By taking, as a starting point of reflection, the recruitment process of vaccine-hesitant parents, this article intends to address the challenges to the research participation of vaccine-hesitant parents. Our arguments thus have relevance for all researchers aiming to enrol hard-to-reach, hidden and vulnerable populations. The current article also responds to Kristensen and Ravn’s (2015) call for recruitment practices to be more deeply discussed in the research community. We believe that through the sharing of experiences, researchers, including us as authors and based on our collaborative efforts with the VAX-TRUST project, will learn from each other (Reich, 2015). We hope that the examples illustrated in this article will be useful for future researchers that intend to conduct research with a hard-to-reach, hidden and vulnerable population such as vaccine-hesitant parents, as well as contribute to the discussion of the need to develop flexible and reflexive approaches to recruitment, when developing this type of research.

## Note

1. For more information on the project, please consult: Cardano M., Numerato D., Gariglio L., Marhánková J., Scavarda A., Bracke P., Hilário A.P., Polak P., Hobson-West P., Vuolanto P. (in progress) A rapid team ethnography on vaccine hesitancy in Europe. Qualitative-midstream protocol paper; Vuolanto P., Almeida A. N., Anderson A., Auvinen P., Beja A., Bracke P., Cardano M., Ceuterick M., Correia T., De Vito E., Delaruelle K., Delicado A., Esposito M., Ferrara M., Cariglio L., Guerreiro C., Marhánková J., Hilario A.P., Hobson-West P., Iorio J., Järvinen K., Kotherova Z., Kuusipalo A., Lermytte E., Mendonça J., Morais R., Numerato D., Polak P., Rudek T., Sbaragli S., Scavarda A., Silva, Katielle S., Silva, P. A., Sivelä J., Soares E., Świątkiewicz-Mośny M., Tipaldo G., Wagner A. (in progress) Trust matters: Towards a social scientific understanding of vaccine hesitancy and the role of healthcare professionals.
